# Prognostic Significance of B7-H3 Expression and CD163+ Tumor-Associated Macrophage Infiltration in Mesothelioma

**DOI:** 10.3390/ijms27156961

**Published:** 2026-08-03

**Authors:** Tülay Koç, Ramazan Oğuz Yüceer, Tuncay Altay, Neslihan Taş, Serkan Çelikgün

**Affiliations:** 1 Pathology Department, Faculty of Medicine, Sivas Cumhuriyet University, 58140 Sivas, Turkey; r.yuceer66@hotmail.com (R.O.Y.); aaltaytuncay@gmail.com (T.A.); 2 Pulmonology Department, Faculty of Medicine, Sivas Cumhuriyet University, 58140 Sivas, Turkey; dr.neslihan.tas@gmail.com; 3 Public Health Department, Faculty of Medicine, Sivas Cumhuriyet University, 58140 Sivas, Turkey; celikgunserkan@gmail.com

**Keywords:** mesothelioma, B7-H3, CD276, CD163, tumor-associated macrophages, tumor microenvironment, immune checkpoint, prognosis

## Abstract

Mesothelioma is a rare, aggressive malignancy with poor prognosis. B7-H3 (CD276), an immune checkpoint molecule, and CD163-positive tumor-associated macrophages (TAMs) contribute to tumor progression and immune evasion. This study evaluated the prognostic impact of B7-H3 expression and CD163-positive TAM infiltration. This single-center retrospective study included 94 patients diagnosed with malignant mesothelioma between 2011 and 2024. B7-H3 expression was assessed using the H-score method and dichotomized at the cohort median. CD163-positive TAM density was quantified in hotspot high-power fields. Overall survival (OS) was analyzed using Kaplan–Meier, log-rank, and multivariable Cox regression analyses. Median OS was 10.15 months (IQR: 4.88–19.66). High B7-H3 expression was associated with shorter OS, whereas high CD163-positive TAM density was associated with longer OS. In multivariable analysis, B7-H3 expression, CD163 expression, tumor localization, recurrence status, and treatment status were independently associated with overall survival. Combined biomarker analysis showed the worst OS in the CD163-low/B7-H3-high group and the best OS in the CD163-high/B7-H3-low group. High B7-H3 expression was independently associated with shorter overall survival, whereas high CD163-positive TAM infiltration was associated with longer overall survival in this cohort. The combined B7-H3/CD163 profile may identify distinct prognostic subgroups and highlights the tumor immune microenvironment.

## 1. Introduction

Mesothelioma is a malignant neoplasm that can arise in any anatomical site lined by a normal mesothelial cell layer, including the pleura, peritoneum, pericardium, and tunica vaginalis [1]. It most commonly involves the visceral pleura, with the peritoneum being the second most frequent site [2]. Pleural mesothelioma (PM) is a rare, aggressive malignancy that is frequently associated with occupational asbestos exposure, and its incidence is increasing worldwide [3]. Peritoneal mesothelioma (PeM) typically presents with diffuse, extensive involvement throughout the peritoneal cavity, and metastatic spread beyond the abdomen is uncommon. Owing to its rarity and nonspecific clinical manifestations, diagnosis is often delayed until the disease is advanced. Because pleural mesothelioma is more common than PeM, most research has focused on the pleural variant, and findings have frequently been extrapolated to PeM. Although therapeutic advances have been achieved, the disease remains lethal, largely due to abdominal complications related to extensive peritoneal dissemination. Without treatment, life expectancy is less than one year. Cytoreductive surgery combined with heated intraperitoneal chemotherapy constitutes the cornerstone of therapy, whereas systemic treatment strategies are still evolving [2].

Despite its aggressive clinical course, the therapeutic landscape of malignant pleural mesothelioma has evolved in recent years. The MAPS phase III trial demonstrated that the addition of the anti-VEGF antibody bevacizumab to cisplatin plus pemetrexed improved overall survival compared with chemotherapy alone [4]. More recently, the CheckMate 743 trial established first-line dual immune checkpoint inhibition with nivolumab plus ipilimumab as an effective treatment option for unresectable malignant pleural mesothelioma, with a particularly pronounced survival benefit in patients with non-epithelioid histology [5]. Nevertheless, long-term survival remains limited, highlighting the need for additional prognostic and therapeutically relevant biomarkers.

Both diffuse and localized mesotheliomas are classified into epithelioid, biphasic, or sarcomatoid subtypes according to the World Health Organization classification. Histologic subtype correlates with prognosis: the sarcomatoid subtype has the worst outcome, followed by the biphasic and epithelioid subtypes. In untreated cases, the median overall survival (OS) is approximately 11 months for the epithelioid subtype, 10 months for the biphasic subtype, and 5 months for the sarcomatoid subtype. After treatment, median OS is reported as 16.9 months for epithelioid, 13.1 months for biphasic, and 5.5 months for sarcomatoid mesothelioma. In a comparative phase III trial evaluating cisplatin plus pemetrexed in previously untreated, unresectable pleural mesothelioma, the median survival was only 12.1 months, underscoring the need for more effective therapeutic agents [3].

B7-H3 (CD276) is an immune checkpoint molecule belonging to the B7 family and is aberrantly expressed in tumor cells as a co-inhibitory ligand that promotes tumor progression. Within the tumor microenvironment (TME), B7-H3 facilitates tumor progression by impairing T-cell responses, promoting polarization of tumor-associated macrophages (TAMs) toward an M2 phenotype, and suppressing the function of other immune cell subsets. In addition, through immune-independent mechanisms, B7-H3 further drives tumor progression by enhancing tumor cell proliferation, migration, invasion, metabolic reprogramming, angiogenesis, and resistance to therapy. Immunotherapeutic strategies targeting B7-H3, including combinations with other immune checkpoint inhibitors, have demonstrated promising antitumor activity [6].

Macrophages are key components of the mesothelioma microenvironment and may have prognostic significance. Tumor-associated macrophages are broadly classified into antitumor M1 and protumor M2 phenotypes, the latter characterized by CD163 expression, cytokine secretion, and immunosuppressive activity. In pleural mesothelioma, M2 macrophages are abundant in both pleural effusions and tumor tissue and may contribute to tumor progression by suppressing CD8+ T-cell function through arginase and cytokine release [7]. In malignant pleural mesothelioma, immune cell infiltration patterns, including macrophage subtypes, have been linked to patient survival outcomes. Specifically, high densities of CD163+ TAMs alone do not consistently correlate with survival; however, their prognostic significance emerges when considered in combination with lymphocyte infiltration. For example, patients with high CD163+ macrophage infiltration and low CD8+ cytotoxic T lymphocyte presence exhibit worse prognosis compared to other groups, while those with low CD163+ and high CD20+ B lymphocyte infiltration show better survival outcomes. Multivariate analyses identify the ratios of CD163 to CD8 and CD163 to CD20 as independent prognostic factors, highlighting the interplay between these immune components in the MPM tumor microenvironment [8].

Macrophages also participate in the early response to asbestos exposure, as asbestos-induced macrophage activation promotes TNF-α secretion, which in turn activates NF-κB signaling in mesothelial cells via TNF receptor I, enhancing cell survival and resistance to cytotoxic injury. This mechanism may facilitate malignant transformation. In addition, macrophages (CD68+) and natural killer cells (CD56+) constitute major components of the inflammatory infiltrate in pleural mesothelioma [7].

Burt et al. demonstrated that a high preoperative monocyte count was negatively correlated with overall survival (OS) in both epithelioid and non-epithelioid mesotheliomas. A higher density of TAMs, compared with a lower TAM density, was significantly associated with worse prognosis in the non-epithelioid group; this association was not observed in the epithelioid group. In addition, expression of CD68+ stromal macrophages was found to correlate with the number of stromal regulatory T cells, suggesting that M2 macrophages may modulate adaptive immune responses [9].

Overall, CD163+ TAM infiltration, particularly when viewed as a proportion of total macrophages or combined with lymphocyte infiltration patterns, constitutes a significant prognostic marker in mesothelioma. High CD163+ macrophage presence, especially alongside low cytotoxic T cell infiltration, predicts poorer survival outcomes. These findings highlight the complex immunosuppressive landscape of the mesothelioma microenvironment and suggest that therapeutic strategies targeting macrophage polarization or modulating immune checkpoints may have prognostic and therapeutic value in this malignancy [8,9,10].

Mesothelioma is an aggressive malignancy—particularly in pleural and peritoneal locations—with limited diagnostic and therapeutic options and persistently poor survival outcomes. Despite currently available systemic treatments, improvements in patient outcomes remain modest. This highlights an unmet need for novel prognostic biomarkers that better reflect tumor biology and for targeted therapeutic strategies. Therefore, further studies capable of providing alternative approaches in this field are warranted.

The primary aim of this study is to investigate the association between B7-H3 (CD276) expression levels and the density of CD163+ TAMs in pleural and peritoneal mesothelioma and to evaluate the prognostic impact of these two biomarkers on clinicopathologic characteristics and overall survival.

## 2. Results

### 2.1. Demographic and Clinical Characteristics

A total of 94 patients were included in this study. The median age was 66 years (IQR: 59–73), with 38 female (40.4%) and 56 male (59.6%) patients. The median overall survival (OS) was 10.15 months (IQR: 4.88–19.66). Asbestos exposure was identified in 85.1% of patients (*n* = 80), while 14.9% (*n* = 14) had no history of exposure. Of the 94 patients, 72 (76.6%) had pleural mesothelioma and 22 (23.4%) had peritoneal mesothelioma. Owing to the marked imbalance in subgroup sizes, formal site-specific analyses of B7-H3 and CD163 were not performed. Demographic and clinical characteristics are summarized in Table 1.

### 2.2. Histopathological Findings and Tumor Grading

Histopathological subtype evaluation revealed a predominance of the epithelioid subtype, accounting for 84.0% of cases (*n* = 79). Biphasic and sarcomatoid subtypes were observed in 12.8% (*n* = 12) and 3.2% (*n* = 3) of patients, respectively. Tumor-infiltrating lymphocytes (TILs) were absent in 33.0% of patients (*n* = 31), mild in 57.4% (*n* = 54), and marked in 9.6% (*n* = 9) (Table 1).

Tumor grading was performed exclusively in the epithelioid subgroup. Nuclear atypia was mild in 16.5% (*n* = 13), moderate in 54.4% (*n* = 43), and severe in 29.1% (*n* = 23) of cases. Mitotic activity was low in 30.4% (*n* = 24), moderate in 57.0% (*n* = 45), and high in 12.7% (*n* = 10). Tumor necrosis was present in 25.3% (*n* = 20) and absent in 74.7% (*n* = 59). Nuclear grade 2 was the most frequent category (57.0%, *n* = 45), followed by grade 1 (30.4%, *n* = 24) and grade 3 (12.7%, *n* = 10). Overall tumor grade was classified as low in 70.9% (*n* = 56) and high in 29.1% (*n* = 23) of cases. Histopathological parameters are presented in Table 2.

### 2.3. Treatment and Clinical Course

During follow-up, recurrence developed in 7.4% of patients (*n* = 7), while 92.6% (*n* = 87) remained recurrence-free. At the end of follow-up, 93.6% of patients (*n* = 88) had died and 6.4% (*n* = 6) were alive. Treatment was administered to 61.7% of patients (*n* = 58), whereas 38.3% (*n* = 36) received no treatment. Among treated patients, chemotherapy was the most common modality (58.6%, *n* = 34), followed by chemotherapy plus surgery (19.0%, *n* = 11), chemotherapy plus radiotherapy (12.1%, *n* = 7), and surgery alone (10.3%, *n* = 6) (Table 1).

Treatment data were available only as broad treatment categories. Detailed regimen-, procedure-, line-, and intent-specific analyses could not be performed because these data were not consistently documented across the retrospective study period.

### 2.4. Survival Analysis

**Figure 1:**
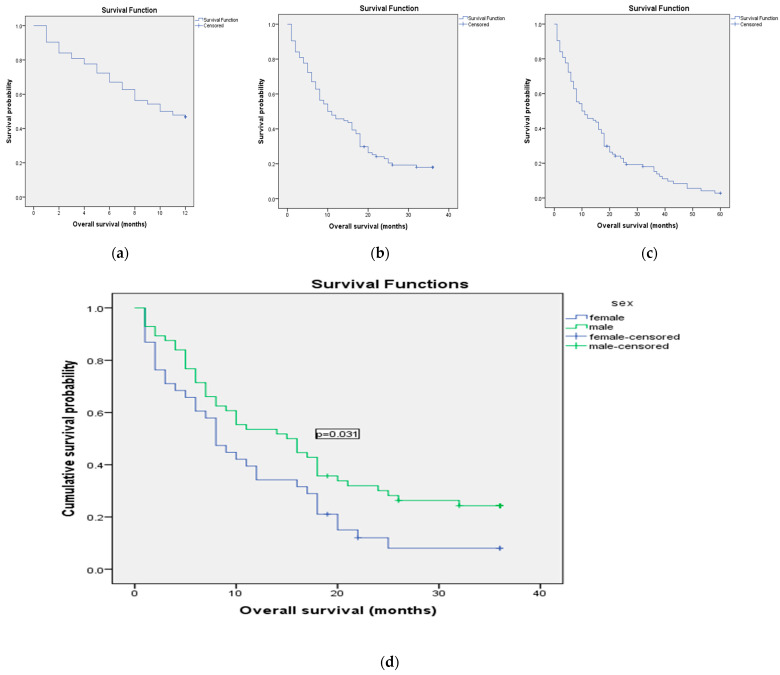

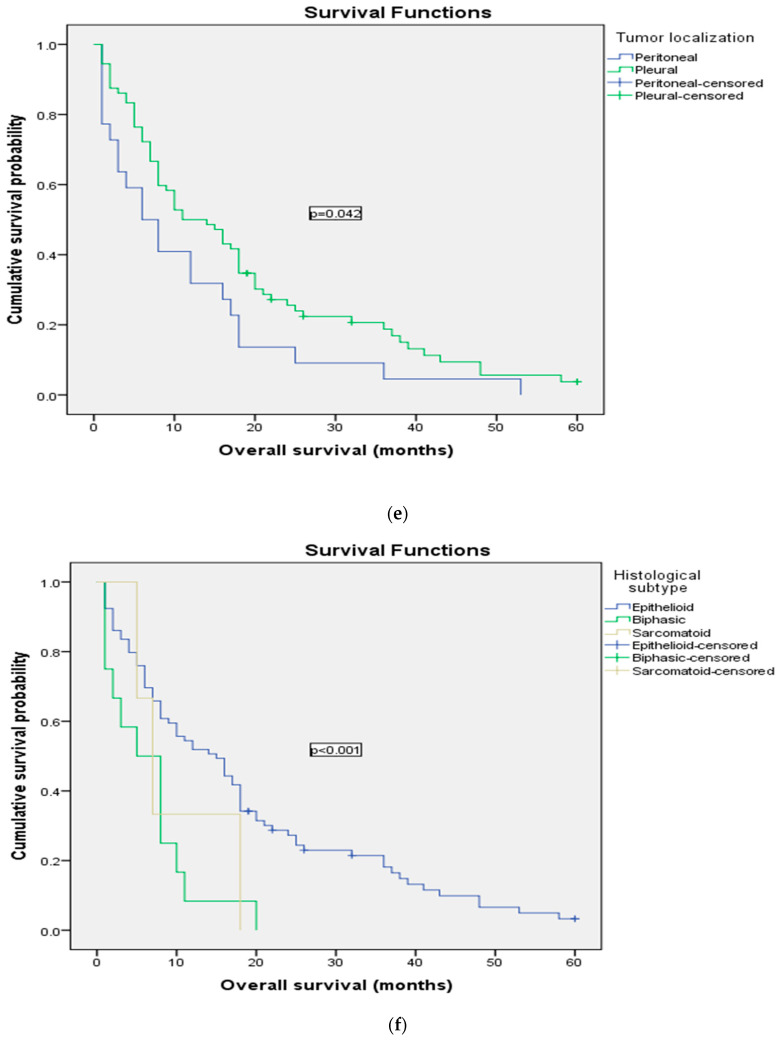

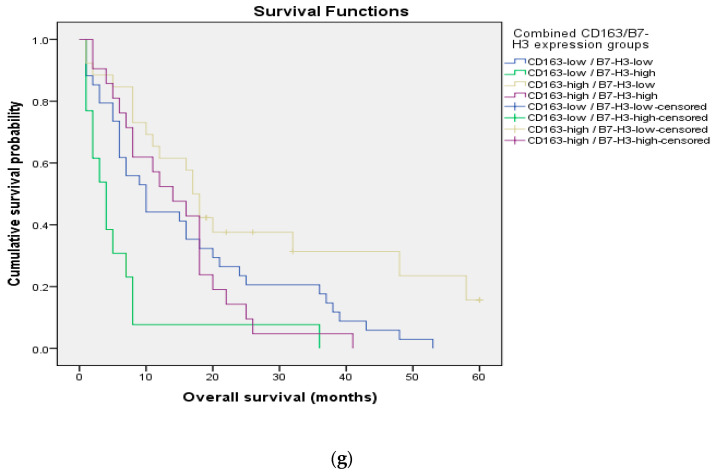
(**a**) Kaplan–Meier curve depicting 12-month overall survival among patients with mesothelioma. (**b**) Kaplan–Meier curve depicting 36-month overall survival among patients with mesothelioma. (**c**) Kaplan–Meier curve depicting 60-month overall survival among patients with mesothelioma. (**d**) Kaplan–Meier curves for 36-month overall survival according to sex. (**e**) Kaplan–Meier curves for 5-year overall survival according to tumor localization. (**f**) Kaplan–Meier curves for 5-year overall survival according to histological subtype. (**g**) Kaplan–Meier curves for 5-year overall survival according to combined CD163 and B7-H3 expression status.

When survival duration was categorized, 53.2% of patients (*n* = 50) died within the first year. The 1-year, 3-year, and 5-year OS rates were 46.8% (*n* = 44), 19.1% (*n* = 18), and 7.4% (*n* = 7), respectively. The mean survival time for the entire cohort was 15.73 ± 1.51 months (95% CI: 12.72–18.74) based on the 5-year survival analysis (Figure 1a–f).

### 2.5. Impact of Immunohistochemical Markers on Survival

**Figure 2:**
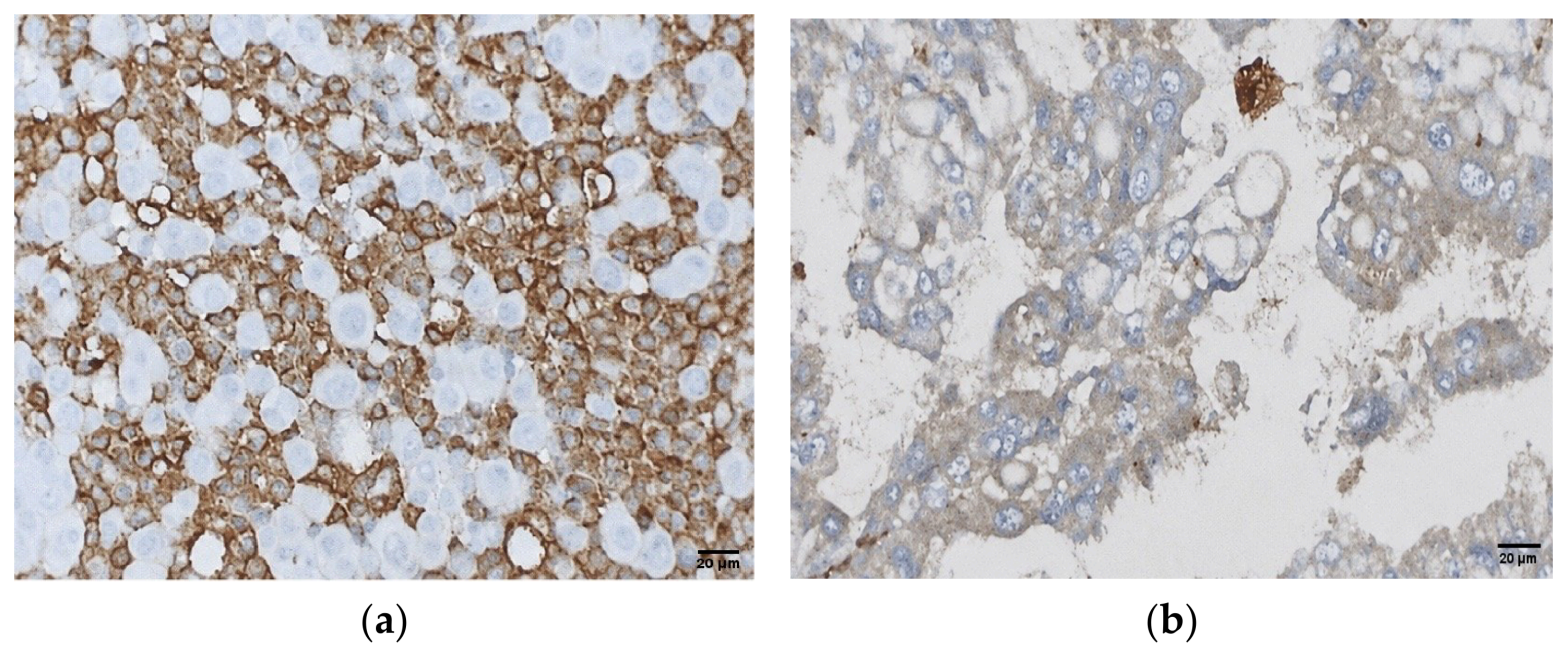
(**a**) CD163 high expression (×400) (**b**) CD163 low expression (×400).

**Figure 3:**
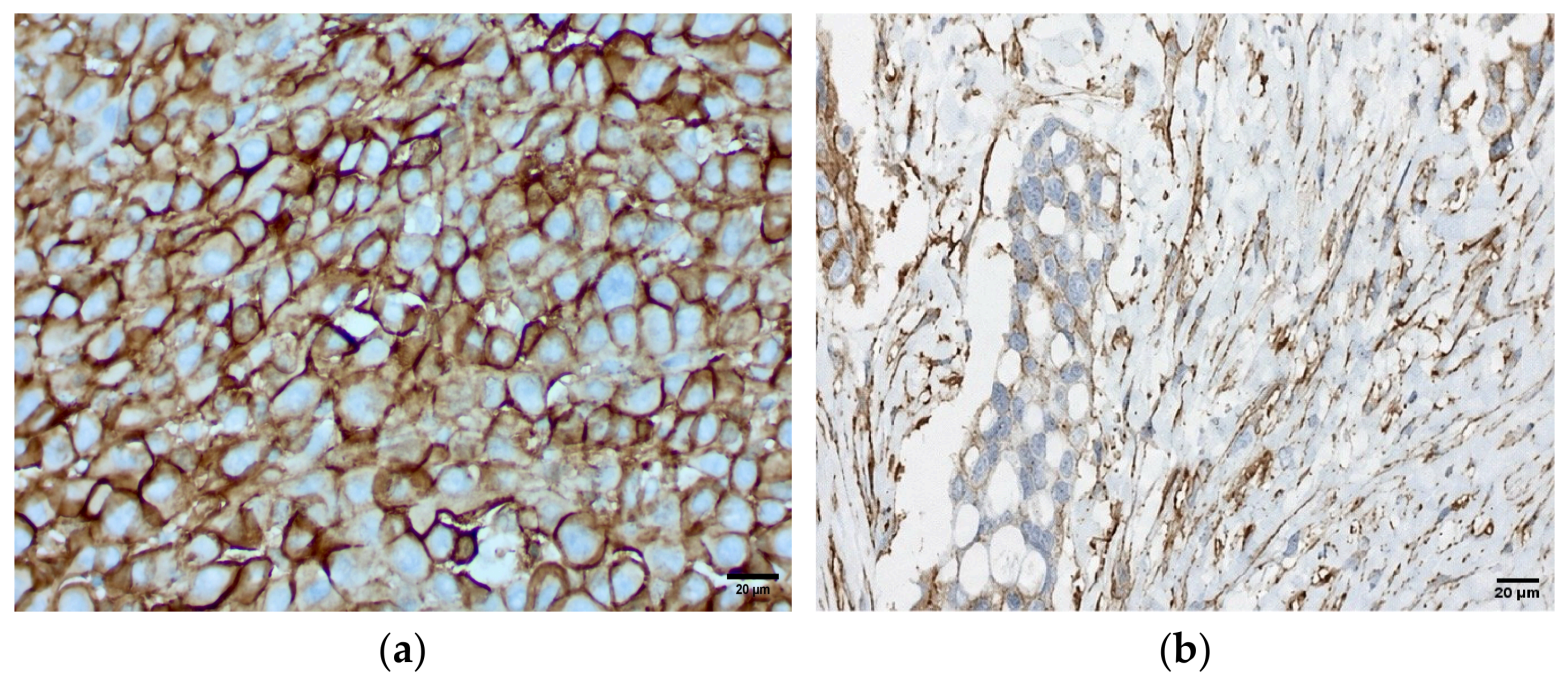
(**a**) B7-H3 high expression (×400) (**b**) B7-H3 low expression (×400).

Immunohistochemical analysis revealed that patients were equally distributed between low and high CD163 expression groups (Figure 2a,b). B7-H3 expression was low in 63.8% of patients (*n* = 60) and high in 36.2% (*n* = 34) (Table 3) (Figure 3a,b).

CD163 expression was significantly associated with OS (*p* = 0.018), with longer survival observed in the high-expression group. B7-H3 expression also showed a significant association with survival (*p* = 0.007); patients with low B7-H3 expression demonstrated superior survival outcomes, suggesting that high B7-H3 expression may serve as an adverse prognostic indicator (Table 3).

### 2.6. Multivariable Cox Regression Analysis

Multivariable Cox regression analysis was performed to evaluate factors independently associated with overall survival. Tumor localization, recurrence status, treatment status, CD163 expression, and B7-H3 expression were all significantly associated with survival in the multivariable model.

Tumor localization was independently associated with overall survival (adjusted HR = 0.456, 95% CI: 0.23–0.87; *p* = 0.019), with a lower hazard of death observed in patients with pleural tumors compared with those with peritoneal tumors. Recurrence status was also significantly associated with overall survival (HR = 0.271, 95% CI: 0.098–0.74; *p* = 0.012). Unexpectedly, patients with recurrence had a lower hazard of death compared with those without recurrence.

Treatment status showed one of the strongest associations with overall survival in the multivariable model (HR = 0.420; *p* = 0.001); treated patients had an approximately 58% lower hazard of death. CD163 expression was independently associated with OS (HR = 0.405; *p* = 0.001), and high CD163 expression was associated with a lower risk of death, suggesting an important role for the immune microenvironment in prognosis. High B7-H3 expression was independently associated with a higher hazard of death in the multivariable model.

In the multivariable model, no statistically significant associations were found between OS and sex, age, asbestos exposure, histopathological subtype, nuclear atypia, mitotic activity, necrosis, or tumor grade (*p* > 0.05), suggesting that the independent prognostic effects of these variables may be limited despite their significance in univariate analyses.

Overall, survival rates in this cohort declined markedly after the first year, with 3- and 5-year OS rates remaining very low, consistent with the aggressive clinical course of malignant mesothelioma. Histopathological features (subtype, mitotic activity, necrosis, and tumor grade), treatment status, and immunohistochemical markers appeared to be the primary determinants of survival, whereas the influence of demographic variables was limited. In particular, absence of treatment, high B7-H3 expression, low CD163 expression, and adverse histopathological parameters were associated with poorer survival. Recurrence status was also significantly associated with overall survival; however, patients with recurrence unexpectedly showed a lower hazard of death (Table 4).

### 2.7. Correlation and Combined Groups

When CD163 and B7-H3 expression patterns were evaluated jointly, the four combined expression groups showed significantly different overall survival distributions (log-rank *p* < 0.001) (Table 5 and Figure 1g). The CD163-high/B7-H3-low group demonstrated the most favorable survival outcome, with a mean overall survival of 26.49 months (95% CI: 17.85–35.14) and a 5-year survival rate of 26.9%. In contrast, the CD163-low/B7-H3-high group had the poorest survival outcome, with a mean overall survival of 6.30 months (95% CI: 1.26–11.35) and no patients surviving at 5 years. The CD163-low/B7-H3-low and CD163-high/B7-H3-high groups showed intermediate mean survival times of 16.11 and 14.33 months, respectively. These findings suggest that the survival advantage associated with high CD163 expression was most evident in tumors with low B7-H3 expression, whereas the combination of low CD163 and high B7-H3 expression identified the subgroup with the least favorable outcome (Table 5).

## 3. Discussion

Mesothelioma is a rare, aggressive malignancy with a poor prognosis and limited treatment options [3]. Therefore, studies are needed to reveal new treatment recommendations for mesothelioma. Differences in survival are associated with age at diagnosis, gender, health status, and tumor- and environment-related factors. In this study, it is noteworthy that survival rates decreased significantly, especially after the first year, and three- and five-year survival rates were quite low. This is consistent with the aggressive course of the disease and suggests limited long-term survival.

Several studies have shown that asbestos exposure is negatively correlated with the prognosis of patients with malignant mesothelioma, and therefore the prognosis can vary across regions with different histories of industrial exposure to asbestos [11]. In this study, a history of asbestos exposure was present in the majority of cases. The better survival observed in patients without asbestos exposure suggests that asbestos exposure may influence prognosis through its effects on disease biology, tumor burden, or concomitant pulmonary damage.

TILs could be a useful indicator for predicting prognosis and guiding personalized treatment strategies in patients with PeM [12]. In this study, the lack of a significant correlation between TIL levels and survival suggests that assessing lymphocyte infiltration solely based on density may be insufficient. The functional status of TILs, their subtypes, intratumor location, and accompanying immune checkpoint expression may be more decisive in determining survival.

Treatment status showed a strong independent association with overall survival in our cohort. However, this finding should be interpreted cautiously because treatment allocation was not randomized and may have been influenced by performance status, disease extent, tumor burden, comorbidities, tumor localization, and other clinical factors. Moreover, detailed information on chemotherapy regimens, surgical procedures, radiotherapy protocols, treatment line and intent, immunotherapy, anti-angiogenic therapy, and cytoreductive surgery/HIPEC was not consistently available. Therefore, selection bias and residual confounding cannot be excluded, and the observed association should not be interpreted as evidence of a causal treatment effect or the efficacy of a specific treatment modality.

B7-H3 belongs to the B7 superfamily of type I transmembrane proteins, which modulate T-cell function in a costimulatory or coinhibitory manner [13,14]. In normal tissues, B7-H3 is generally expressed at low levels. It may also be detected in several immune cell populations, including monocytes, dendritic cells, myeloid-derived suppressor cells, neutrophils, macrophages, B cells, and activated T cells. In contrast, B7-H3 is aberrantly overexpressed in many malignancies, including non-small cell lung cancer, and has been associated with poor prognosis in several tumor types [13,15]. However, the molecular mechanisms underlying the correlation between B7-H3 expression and poor prognosis have not been completely elucidated, and the effect of B7-H3 on the cancer–immune system interactions is controversial. Although B7-H3 was initially identified as an activator of T-cell function, subsequent reports have shown that B7-H3 can cause T-cell dysfunction. The receptor for B7-H3 has not been identified yet, but the crystal structure of mouse B7-H3 has revealed that the FG loop of the IgV domain of B7-H3 engages the receptor of T cells and plays a critical role in T-cell dysfunction. Furthermore, despite the high incidence of B7-H3 expression in malignancies, the immunologic interactions between B7-H3 and PD-L1, which is also preferably expressed in malignancies, are not clear. Studies investigating B7-H3 expression in mesothelioma are scarce [13].

Crispen et al. reported that enhanced tumor expression of B7-H3 correlates with adverse clinical and pathologic features of clear cell renal cell carcinoma and independently predicts disease progression and cancer-specific death [16]. Higher expression of B7-H3 is more frequently observed in patients with metastatic cancer than in those with localized cancer. For example, patients with metastatic prostate cancer, high B7-H3 expression was independently associated with high disease-specific mortality and overall mortality rates [17]. High levels of B7-H3 expression have been found in all the cancer types tested. In a meta-analysis study of 24 observational studies consisting of 4141 patients, an elevated baseline B7-H3 did significantly correlate with poor OS and recurrence-free survival (RFS) across a wide range of tumor types [15]. Enhanced expression of B7-H3 in tumors has been linked with therapeutic resistance, metastasis potential and poor patient prognosis [18].

In contrast to the generally adverse prognostic implications of recurrence, recurrence status in our cohort showed an unexpected inverse association with mortality, with a lower hazard of death observed in patients with documented recurrence. This finding should be interpreted cautiously, particularly because only seven patients had recurrence, resulting in a very small subgroup and potentially unstable effect estimates. In addition, patients must survive long enough for recurrence to occur and be documented, which may introduce survivorship or time-related bias. Therefore, this result should not be interpreted as suggesting a protective effect of recurrence on survival.

Matsumura et al. found that B7-H3 is widely expressed in mesothelioma. Notably, PD-L1 and B7-H3 were frequently co-expressed in tumor cells of the non-epithelioid subtype, where unmet medical needs are particularly high. In contrast, they did not detect PD-L1 or B7-H3 expression in normal mesothelial cells and therefore suggested that B7-H3 may represent a potential target for drug development [3]. Yonesaka et al. reported that B7-H3 expression on tumor cells may facilitate immune evasion, potentially by escaping CD8+ T cell-mediated immune surveillance. They further suggested that anti-B7-H3 immunotherapy, in combination with anti-PD-1/PD-L1 antibody therapy, represents a promising therapeutic strategy for B7-H3-expressing non-small cell lung cancers [13]. In our study, high B7-H3 expression was found to be associated with an increased risk of death in multivariate analysis. This finding suggests that B7-H3 may be a poor prognostic immune checkpoint marker in mesothelioma. It is suggested that B7-H3 may be associated with tumor immune evasion, suppression of T cell response, tumor progression, and metastatic potential. Therefore, high B7-H3 expression may be associated with worse survival as an indicator of an immunosuppressive tumor microenvironment. B7-H3 expression was found to be near the significance level in overall survival analysis, but B7-H3 expression remained independently associated with overall survival after adjustment for the variables included in the multivariable model. This association persisted after adjustment for the clinicopathological variables included in the model; however, causality cannot be inferred from the present observational study.

Our findings regarding B7-H3 should be interpreted as confirmatory rather than entirely novel. Previous studies have demonstrated frequent CD276 (B7-H3) expression in pleural mesothelioma and have reported associations with adverse clinicopathological features and unfavorable clinical outcomes. In particular, the ETOP Mesoscape Consortium study by Haberecker et al. provided large-scale evidence regarding the prevalence and clinical significance of B7-H3 expression in pleural mesothelioma [19]. The present findings are consistent with these observations, as high B7-H3 expression was associated with a higher hazard of death in our cohort. The main contribution of this study is the combined evaluation of B7-H3 expression and CD163-positive TAM infiltration, rather than the identification of B7-H3 as a previously unrecognized prognostic marker.

The association between high B7-H3 expression and shorter overall survival supports the biological relevance of this immune checkpoint molecule in mesothelioma. However, the present study was observational and immunohistochemical and did not include functional validation, mechanistic experiments, or treatment-response data. Therefore, these findings do not establish B7-H3 as an effective therapeutic target or demonstrate that B7-H3 expression predicts response to targeted therapy. Further preclinical and prospective clinical studies are required to evaluate its therapeutic relevance.

Innate immune cells, and, more specifically, macrophages are involved at an early stage of the immune response following asbestos exposure [7]. Macrophages can be present within the tumor and are called TAMs and may constitute prognostic factors. They can be divided into M1-macrophages with anti-tumor activity and M2-macrophages (CD163+), which are characterized by pro-tumor activity through the secretion of several cytokines (IL-1, IL-6, IL-10, VEGF and TGF-β) [20]. M2-macrophages have been found to be highly present in both pleural effusion and tissue samples of patients with an MPM (100% of patients with M2-macrophage expression > 41% in pleural fluid and 44% in pleural tissue, respectively) [21]. In our study, CD163 expression was associated with better survival and a lower risk of death in the high expression group. This finding suggests that the role of CD163-positive tumor-associated macrophages in the mesothelioma microenvironment may vary depending on tumor type, local immune binding, and concomitant immune cell infiltration.

The association between high CD163-positive TAM density and longer overall survival was an unexpected finding and is not fully aligned with the conventional interpretation of CD163 as a marker of M2-like, immunosuppressive macrophages. In mesothelioma, single-marker CD163+ TAM infiltration has not consistently correlated with survival, whereas prognostic value emerges when CD163 is evaluated alongside other immune markers such as CD8 and CD20 [8]. Likewise, the CD163/CD68 ratio has been reported to correlate with overall survival, even when the absolute number of CD163-positive cells does not [10]. These data suggest that the biological effect of CD163-positive TAMs is context-dependent and may vary with the immune and stromal microenvironment, the spatial compartment assessed, and the counting strategy used. Accordingly, the present result should not be interpreted as evidence that CD163-positive macrophages are intrinsically protective in mesothelioma but rather as a hypothesis-generating finding that warrants validation in larger independent cohorts with multiplex immune profiling and additional macrophage markers [8].

The traditional M1/M2 polarization model provides a useful conceptual framework but does not adequately capture the transcriptional, functional, and spatial heterogeneity of tumor-associated macrophages. Contemporary single-cell and spatial transcriptomic studies have identified multiple macrophage states that extend beyond this binary classification. Among these, SPP1-positive macrophages have been associated with tumor stemness, immune suppression, chemoresistance, and poor clinical outcomes. In liver cancer, SPP1-positive macrophages were shown to promote tumor stemness through vitronectin-dependent signaling and reciprocal CCL15-mediated tumor–macrophage crosstalk [22].

C1QC-positive macrophages represent another distinct macrophage population whose biological effects appear to depend on the surrounding stromal and immune context. Recent evidence indicates that FAP-positive fibroblasts may promote C1QC-positive macrophage infiltration through WNT2 signaling, thereby contributing to T-cell exhaustion in the tumor microenvironment. These observations emphasize that macrophage phenotype and prognostic significance are determined not only by individual markers but also by cellular interactions, activation states, and spatial organization [23].

Therefore, CD163 expression alone should not be regarded as a definitive surrogate for a homogeneous M2-like, protumor macrophage population. The association between high CD163-positive TAM density and improved survival observed in the present study may reflect differences in macrophage subpopulation composition, spatial distribution, or interaction with lymphocytes, fibroblasts, and other stromal elements. Since multiplex immunophenotyping, single-cell profiling, and markers such as SPP1 and C1QC were not evaluated, the biological basis of this association remains uncertain and should be investigated in future studies.

An unexpected finding of the present study was the association between high CD163-positive TAM density and longer overall survival. This result differs from previous reports in malignant pleural mesothelioma, including the study by Fukui et al., which suggested that M2-like tumor-associated macrophages may promote tumor progression and be associated with unfavorable clinical outcomes [24]. Several factors may account for this discrepancy, including differences in tumor site, histological subtype distribution, treatment exposure, tissue sampling, hotspot selection, and the broader immune context. In addition, CD163 alone may not adequately define macrophage functional phenotype, as CD163-positive macrophages represent a heterogeneous population with potentially context-dependent biological effects. Therefore, our finding should not be interpreted as evidence that CD163-positive macrophages are intrinsically protective and requires validation in larger cohorts using broader immune profiling.

In addition to tumor cell-surface immune regulatory proteins, stromal and extracellular matrix components may substantially influence the immune microenvironment of mesothelioma. Versican, for example, has been reported to modulate tumor-associated macrophage properties and promote mesothelioma growth, indicating that stromal signaling can affect both macrophage and lymphocyte responses [25]. These stromal determinants were not assessed in the present study. Therefore, the observed associations between B7-H3 expression, CD163-positive TAM infiltration, and survival should be interpreted within the context of an incompletely characterized tumor microenvironment.

B7-H3 regulates the differentiation of tumor-associated macrophages and promotes the polarization of type 2 macrophages and switching of the M1 phenotype to the M2 phenotype, and B7-H3 also contributes to CCL2–CCR2–M2 macrophage axis-mediated immunosuppression [26].

These findings suggest that B7-H3 may be a potential prognostic marker and therapeutic target in mesothelioma and that CD163-positive macrophage infiltration is a parameter that can be considered in the prognostic classification of the tumor microenvironment.

This study has several important limitations. First, its retrospective, single-center design and relatively small sample size limit the external validity of the findings and increase the risk of selection bias and residual confounding. The cohort included both pleural and peritoneal mesothelioma, which are clinically and biologically distinct diseases with different treatment approaches and prognostic characteristics. In addition, the clinicopathological subgroups were small and markedly unbalanced, particularly with respect to peritoneal localization, biphasic and sarcomatoid histology, recurrence status, and the combined B7-H3/CD163 categories. Consequently, adequately powered subgroup, sensitivity, stratified, and interaction analyses could not be performed, and subgroup-specific estimates may be unstable. Although tumor localization was included in the multivariable model, residual site-related confounding cannot be excluded.

Second, treatment-related analyses were limited by incomplete and non-standardized retrospective data. Treatment allocation was not randomized and may have been influenced by performance status, disease extent, tumor burden, comorbidities, tumor localization, and patient eligibility. Detailed information on standardized disease stage, performance status, tumor burden, chemotherapy regimens, surgery type, radiotherapy dose and intent, treatment line and intent, immunotherapy, anti-angiogenic therapy, and cytoreductive surgery with hyperthermic intraperitoneal chemotherapy was not consistently available. Therefore, treatment-selection bias, immortal-time bias, and residual confounding cannot be excluded, and the observed association between treatment status and survival should not be interpreted as evidence of a causal treatment effect or the efficacy of a specific treatment modality.

Third, B7-H3 and CD163 were dichotomized using cohort-derived median values because no externally validated or clinically established cutoff thresholds are currently available in mesothelioma. Although this approach provided a reproducible basis for exploratory group comparisons, median-based dichotomization is data-dependent, may reduce statistical power, obscure dose–response relationships, and limit external generalizability. Alternative cutoff analyses were not performed because no prespecified validated thresholds were available and the limited sample size increased the risk of unstable and overfitted estimates. In addition, no independent validation cohort was available. Therefore, the proposed cutoff values should not be regarded as clinically established, and the observed associations require external validation.

Fourth, the tumor immune microenvironment was characterized using a limited immunohistochemical panel. CD163 alone does not adequately define macrophage phenotype or capture the transcriptional, functional, and spatial heterogeneity of tumor-associated macrophages, including emerging subpopulations such as SPP1-positive and C1QC-positive macrophages. Complementary macrophage, lymphocyte, and immune checkpoint markers, including CD68, CD8, CD20, FOXP3, PD-1, and PD-L1, were not evaluated. Stromal components, extracellular matrix mediators, spatial immune-cell interactions, and metastatic status were also not systematically assessed. These limitations restrict the biological interpretation of the unexpected association between high CD163-positive TAM density and longer survival and preclude evaluation of the relationships between B7-H3, CD163, other immune checkpoints, and the broader immune context.

Fifth, B7-H3 and CD163 assessments were based on semiquantitative H-score evaluation and hotspot-based cell counting. Although all slides were independently reviewed by two blinded pathologists and discrepancies were resolved by consensus, observer-specific scores were not retained separately; therefore, quantitative interobserver reproducibility could not be assessed using intraclass correlation coefficients or kappa statistics. This may limit the reproducibility of the biomarker measurements.

Finally, the observational and immunohistochemical design of the study does not establish causality, predictive performance, functional mechanisms, or therapeutic relevance. No mechanistic experiments, functional validation, or treatment-response data were available. Accordingly, the associations of B7-H3 and CD163 with survival, including the combined biomarker subgroup findings, should be considered exploratory and hypothesis-generating. Larger, multicenter, site-specific, and independent cohorts using standardized biomarker assessment, broader immune profiling, continuous-variable analyses, validated cutoff values, and comprehensive clinical data are required to confirm these findings.

## 4. Materials and Methods

This single-center retrospective study reviewed the archives of the Department of Pathology Sivas Cumhuriyet University Faculty of Medicine. All cases diagnosed as malignant mesothelioma between 2011 and 2024 were screened.

Inclusion criteria were histopathologic confirmation on H&E supported by appropriate immunohistochemistry, availability of adequate FFPE tissue for additional staining, and accessible clinical follow-up/survival data. Cases with insufficient tissue, significant fixation artifacts, duplicate/repeat samples, or missing key clinical information were excluded. Demographic and clinicopathologic variables (age, sex, tumor site, histologic subtype, vital status, asbestos exposure and follow-up duration) were obtained from the hospital information system.

The diagnosis of malignant mesothelioma was confirmed by integrating histomorphological findings with immunohistochemical results. The diagnostic panel included the mesothelial markers (calretinin, WT1, D2-40 and CK5/6) and the adenocarcinoma markers (claudin-4, Ber-EP4 and/or MOC-31). Additional antibodies, including (BAP1, p53), were used when required according to the morphological and clinical differential diagnosis. All hematoxylin and eosin-stained slides were re-evaluated under light microscopy for nuclear atypia, mitotic activity, necrosis, tumor-infiltrating lymphocytes (TILs), and other morphological features. Stromal TIL density was categorized as absent, mild, or severe according to the extent of lymphocytic infiltration observed on hematoxylin and eosin-stained sections.

Tumors were graded using the WHO Classification of Thoracic Tumors two-tier histopathological grading system. Mitotic activity was assessed in a 2 mm^2^ area in the most active regions. Nuclear atypia was scored as mild, moderate, or severe; mitotic activity was scored as low (≤1), intermediate (2–4), or high (≥5) according to mitoses per 2 mm^2^. Nuclear grades I–III were assigned based on the total score. Overall tumor grade was defined as low grade for nuclear grade I or II without necrosis and high grade for nuclear grade II with necrosis or nuclear grade III, regardless of necrosis.

### 4.1. Tissue Preparation and Immunohistochemistry

Representative FFPE blocks containing adequate viable tumor were selected. Serial 3–4 μm sections were cut and mounted on positively charged slides. Immunohistochemistry for B7-H3 (anti-CD276 antibody [EPR20115], 1:1000 dilution; Abcam, Cambridge, UK) and CD163 (MRQ-26) was performed on a Ventana automated staining platform (Ventana Medical Systems, Tucson, AZ, USA) using standardized protocols. Appropriate positive and negative controls were included in each run.

### 4.2. Evaluation of B7-H3 Expression (H-Score)

B7-H3 expression was evaluated in tumor cells and recorded as membranous and/or cytoplasmic. Semi-quantitative scoring was performed using the H-score method: H-score = Σ (intensity [0–3] × percentage of positive tumor cells), yielding a total score of 0–300. For categorical analyses, cases were classified as low or high expression using the cohort median H-score as the cutoff.

For categorical analyses, B7-H3 expression was dichotomized using the cohort median H-score of 160. The median was selected because no externally validated or clinically established cutoff value is currently available for B7-H3 expression in malignant mesothelioma. This data-driven approach reduced investigator-dependent threshold selection and enabled the formation of reasonably sized comparison groups. Cases with an H-score equal to the median were assigned to the low-expression group.

### 4.3. Assessment of CD163+ Tumor-Associated Macrophages

CD163 staining was used to estimate tumor-associated macrophage (TAM) density in intratumoral and/or stromal areas, excluding necrotic or artifacted regions. Hotspot areas were identified at low magnification, and CD163-positive cells were counted in three hotspot HPFs at ×400; the mean count (cells/HPF) was recorded. TAM density was analyzed as a continuous variable and dichotomized into low/high using the median value as the cutoff.

### 4.4. Combined Biomarker Groups

Cases were classified into four groups according to B7-H3 (low/high) and CD163+ TAM density (low/high): low/low, high/low, low/high, and high/high (median cutoffs).

### 4.5. Interobserver Assessment

All slides were independently scored by two blinded pathologists. Discrepancies were resolved by joint review and consensus.

### 4.6. Survival Endpoint

Overall survival (OS) was defined as the interval from diagnosis to death or last follow-up.

### 4.7. Statistical Analysis

Analyses were performed using SPSS v23.0. Continuous variables were summarized as median (IQR) and categorical variables as *n* (%). Normality was assessed by Shapiro–Wilk. Group comparisons used Mann–Whitney U or Kruskal–Wallis tests for continuous data and chi-square/Fisher’s exact tests for categorical data. Correlation between B7-H3 H-score and CD163 density was assessed by Spearman’s test. OS was analyzed by Kaplan–Meier with log-rank testing. Cox analysis was applied to the significant ones in the logrank test. Cox regression (univariable and multivariable) was used to identify prognostic factors, reporting HRs with 95% CIs; *p* < 0.05 was considered significant.

The study protocol was approved by the Ethics Committee of Sivas Cumhuriyet University Faculty of Medicine (Approval No: 2026-02/69, 26 February 2026).

## 5. Conclusions

In this retrospective cohort, high B7-H3 expression was independently associated with shorter overall survival, whereas high CD163-positive tumor-associated macrophage density was associated with longer overall survival. However, these findings should be interpreted cautiously because of the single-center design, limited sample size, unbalanced subgroups, cohort-derived cutoff values, incomplete clinical data, and restricted characterization of the tumor immune microenvironment.

The combined B7-H3/CD163 analysis should be regarded as exploratory and hypothesis-generating rather than confirmatory. In addition, the present study did not include functional validation, mechanistic experiments, treatment-response analyses, or standardized metastatic-status data. Therefore, the causal, predictive, and therapeutic relevance of B7-H3 and CD163 cannot be established from these findings.

Larger, independent, multicenter studies using standardized immunohistochemical assessment, broader immune profiling, continuous-variable analyses, validated cutoff values, and comprehensive clinical data are required to confirm these associations and clarify their biological and clinical significance.

## Figures and Tables

**Table 1:** Sociodemographic, clinical characteristics and five-year survival analysis. Bold indicates significant differences at *p* < 0.05.

*n* = 94	*n* (%)	Survival Rate *n* (%)	Survival Time (Months) Mean ± 95% CI	*p*-Value
**Sex**				
** Female**	38 (40.4)	3 (7.9)	15.52 ± 2.23 (8.14–16.89)	0.065
** Male**	56 (59.6)	4 (7.1)	19.40 ± 2.26 (14.96–23.84)	
**Age**				
** 0–64**	40 (42.6)	3 (7.5)	19.51 ± 2.64 (14.32–24.70)	0.157
** 65 and above**	54 (57.4)	4 (7.4)	14.49 ± 2.07 (10.43–18.55)	
**Asbestos exposure**				
** Yes**	80 (85.1)	4 (5.0)	15.20 ± 1.73 (11.81–18.59)	0.065
** No**	14 (14.9)	3 (21.4)	25.02 ± 4.46 (16.28–33.77)	
**Localization**				
** Peritoneal**	22 (23.4)	0 (0.0)	14.45 ± 2.77 (6.00–16.90)	**0.042**
** Pleural**	72 (76.6)	7 (9.7)	18.27 ± 1.96 (14.42–22.12)	
**Subtypes**				
** Epithelioid**	79 (84.0)	7 (8.9)	18.48 ± 1.88 (14.78–22.18)	**0.004**
** Biphasic**	12 (12.8)	0 (0.0)	6.50 ± 1.62 (3.32–9.67)	
** Sarcomatoid**	3 (3.2)	0 (0.0)	10.00 ± 4.04 (2.07–17.92)	
**Epithelioid (%)**				
** 10**	1 (1.1)	0 (0.0)	1.00 ± 0.00 (1.00–1.00)	**<0.001**
** 30**	1 (1.1)	0 (0.0)	1.00 ± 0.00 (1.00–1.00)	
** 50**	9 (9.6)	0 (0.0)	7.22 ± 1.90 (3.48–10.95)	
** 80**	1 (1.1)	0 (0.0)	11.00 ± 0.00 (11.00–11.00)	
** 100**	79 (84.0)	7 (8.9)	18.48 ± 1.88 (14.78–22.18)	
**Sarcomatoid (%)**				
** 20**	1 (1.1)	0 (0.00)	11.00 ± 0.00 (11.00–11.00)	**<0.001**
** 50**	9 (9.6)	0 (0.00)	7.22 ± 1.90 (3.48–10.95)	
** 70**	1 (1.1)	0 (0.00)	1.00 ± 0.00 (1.00–1.00)	
** 90**	1 (1.1)	0 (0.00)	1.00 ± 0.00 (1.00–1.00)	
** 100**	3 (3.2)	0 (0.00)	10.00 ± 4.04 (2.07–17.92)	
**TIL**				
** No**	31 (33.0)	0 (0.0)	13.71 ± 2.61 (8.59–18.82)	0.345
** Mild**	54 (57.4)	6 (11.1)	17.97 ± 2.27 (13.51–22.42)	
** Severe**	9 (9.6)	1 (11.1)	18.88 ± 5.16 (8.77–29.00)	
**Therapy**				
** No**	36 (38.3)	0 (0.0)	9.22 ± 1.91 (5.46–12.98)	**<0.001**
** Yes**	58 (61.7)	7 (12.1)	21.23 ± 2.19 (16.92–25.53)	
**Surgery (S)**	6 (10.3)	0 (0.0)	14.33 ± 5.15 (4.22–24.43)	0.066
**Chemotherapy (CT)**	34 (58.6)	3 (8.8)	17.52 ± 2.60 (12.42–22.62)	
**CT + S**	11 (19.0)	1 (9.1)	29.48 ± 4.76 (20.14–38.82)	
**Radiotherapy + CT**	7 (12.1)	3 (42.9)	33.28 ± 8.24 (17.12–49.43)	
**Recurrence**				
** No**	87 (92.6)	7 (8.0)	15.44 ± 1.69 (12.11–18.77)	0.078
** Yes**	7 (7.4)	0 (0.00)	32.42 ± 5.05 (22.51–42.33)	

**Table 2:** Grading results in epithelioid mesothelioma. Bold indicates significant differences at *p* < 0.05.

*n* = 79	*n* (%)	Survival Rate *n* (%)	Survival Time (Months) Mean ± 95% CI	*p*-Value
**Nuclear atypia**				
** Mild**	13 (16.5)	2 (15.4)	26.92 ± 5.15 (16.83–37.01)	**0.034**
** Moderate**	43 (54.4)	5 (11.6)	19.29 ± 2.63 (14.12–24.45)	
** Severe**	23 (29.1)	0 (0.0)	12.30 ± 2.64 (7.11–17.49)	
**Mitosis**				
** Low**	24 (30.4)	4 (16.7)	24.46 ± 3.96 (16.68–32.24)	**0.003**
** Intermediate**	45 (57.0)	3 (6.7)	17.67 ± 2.30 (13.16–22.18)	
** High**	10 (12.7)	0 (0.00)	7.50 ± 2.42 (2.74–12.25)	
**Necrosis**				
** No**	59 (74.7)	7 (11.9)	21.99 ± 2.31 (17.46–26.53)	**<0.001**
** Yes**	20 (25.3)	0 (0.00)	8.30 ± 1.55 (5.26–11.34)	
**Nuclear grade**				
** Grade1 (score 2–3)**	24 (30.4)	4 (16.7)	24.46 ± 3.96 (16.68–32.24)	**0.003**
** Grade2 (score 4–5)**	45 (57.0)	3 (6.7)	17.67 ± 2.30 (13.16–22.18)	
** Grade3 (score 6)**	10 (12.7)	0 (0.0)	7.50 ± 2.42 (2.74–12.25)	
**Tumor Grade**				
** Low**	56 (70.9)	7 (12.5)	22.63 ± 2.39 (17.92–27.33)	**<0.001**
** High**	23 (29.1)	0 (0.0)	8.65 ± 1.53 (5.63–11.67)	

**Table 3:** 5-year survival analysis with CD163 and B7-H3 expression results. Bold indicates significant differences at *p* < 0.05.

	*n* (%)	Survival Rate *n* (%)	Survival Time (Months) Mean ± 95% CI	*p*-Value
**CD163 (median = 45)**				
** Low**	47 (50.0)	0 (0.0)	13.40 ± 2.09 (9.29–17.51)	**0.018**
** High**	47 (50.0)	7 (14.9)	20.43 ± 1.65 (13.42–19.92)	
**B7-H3 (median = 160)**				
** Low**	60 (63.8)	7 (11.7)	19.91 ± 2.35 (15.30–24.51)	**0.007**
** High**	34 (36.2)	0 (0.0)	11.26 ± 1.73 (7.86–14.66)	

**Table 4:** Factors Associated with Five-Year Survival in the Research Group; Results of Cox Regression Analysis. Bold indicates significant differences at *p* < 0.05.

Variables	HR	(95% CI)	*p*-Value
**Age**	0.99	0.97–1.03	0.903
**Sex**			
** Female**	1.00		
** Male**	0.64	0.37–1.09	0.107
**Asbestos exposure**			
** Yes**	1.00		
** No**	1.08	0.53–2.19	0.824
**Localization**			
** Peritoneal**	1.00		
** Pleural**	0.45	0.23–0.87	**0.019**
**Histological subtype**			
** Epithelioid**	1.00		
** Biphasic**	0.94	0.35–2.54	0.911
** Sarcomatoid**	0.70	0.16–3.06	0.644
**Mitosis**			
** Low**	1.00		
** Moderate**	1.04	0.24–4.52	0.954
** High**	1.02	0.34–3.03	0.965
**Necrosis**			
** No**	1.00		
** Yes**	0.69	0.26–1.83	0.458
**Tumor grade**			
** Low**	1.00		
** High**	0.35	0.10–1.25	0.108
**Recurrence**			
** No**	1.00		
** Yes**	0.27	0.098–0.74	**0.012**
**Therapy**			
** No**	1.00		
** Yes**	0.42	0.24–0.71	**0.001**
**CD163**			
** Low**	1.00		
** High**	0.40	0.23–0.69	**<0.001**
**B7-H3**			
** Low**	1.00		
** High**	1.85	1.06–3.23	**0.030**

**Table 5:** Relationship of combined groups with survival. Bold indicates significant differences at *p* < 0.05.

Groups	*n* (%)	Survival Rate (%)	Survival Time (Months) Mean ± 95% CI	*p*-Value
**CD163 low/B7-H3 low**	34 (36.2)	0 (0.0)	16.11 ± 2.59 (11.02–21.20)	**<0.001**
**CD163 low/B7-H3 high**	13 (13.8)	0 (0.0)	6.30 ± 2.57 (1.26–11.35)	
**CD163 high/B7-H3 low**	26 (27.7)	7 (26.9)	26.49 ± 4.41 (17.85–35.14)	
**CD163 high/B7-H3 high**	21 (22.3)	0 (0.0)	14.33 ± 2.08 (10.24–18.42)	

## Data Availability

The data presented in this study are available on request from the corresponding author. The data are not publicly available due to privacy and ethical restrictions related to patient confidentiality.

## Abbreviations

CI	Confidence interval
CT	Chemotherapy
CTLA-4	Cytotoxic T-lymphocyte-associated protein 4
HR	Hazard ratio
IHC	Immunohistochemistry
IL	Interleukin
IQR	Interquartile range
MDSC	Myeloid-derived suppressor cell
PeM	Peritoneal mesothelioma
PM	Pleural mesothelioma
NK	Natural killer (cell)
OS	Overall survival
PD-1	Programmed cell death protein 1
PD-L1	Programmed death-ligand 1
SD	Standard deviation
SE	Standard error
TAM	Tumor-associated macrophage
TGF-β	Transforming growth factor beta
TME	Tumor microenvironment
VEGF	Vascular endothelial growth factor
WHO	World Health Organization
